# From Regenerative to Degenerative Niche: Multicellular Crosstalk and Population Dynamics in Skeletal Muscle Aging

**DOI:** 10.1111/acel.70733

**Published:** 2026-09-29

**Authors:** Seongwan Kim, Min Ju Kim, Yong Ryoul Yang

**Affiliations:** ^1^ Aging Convergence Research Center Korea Research Institute of Bioscience and Biotechnology (KRIBB) Daejeon Republic of Korea; ^2^ Department of Bio‐Molecular Science, KRIBB School of Bioscience Korea University of Science and Technology (UST) Daejeon Republic of Korea

**Keywords:** aging, cellular senescence, fibro‐adipogenic progenitors, immune remodeling, intercellular communication, muscle stem cells, neuromuscular junction, sarcopenia, skeletal muscle aging

## Abstract

Skeletal muscle aging is increasingly recognized as a failure of tissue‐level coordination rather than a consequence of isolated defects in individual cell types. Recent advances in single‐cell, spatial, and multimodal omics have revealed that aging remodels the abundance, functional states, and interactions of muscle‐resident populations, shifting the tissue from a regenerative niche toward a degenerative niche. In this Review, we summarize current evidence supporting this conceptual transition by focusing on multicellular crosstalk and population dynamics within the aging muscle microenvironment. We discuss how age‐dependent remodeling of muscle stem cells, fibro‐adipogenic progenitors, immune cells, vascular cells, and neuromuscular components collectively disrupts the temporal coordination required for effective regeneration. Rather than acting independently, these populations become locked in maladaptive signaling circuits that promote persistent inflammation, fibrosis, senescence, impaired vascular support, and neuromuscular dysfunction, ultimately compromising tissue repair and muscle function. We further distinguish ligand‐receptor interactions inferred from single‐cell atlases from signaling pathways that have been functionally validated in vivo, highlighting the importance of establishing causal mechanisms underlying intercellular communication. Finally, we discuss emerging therapeutic strategies aimed at restoring multicellular coordination—including modulation of stromal, immune, vascular, and neuromuscular interactions—rather than targeting single cell populations in isolation. We propose that rebuilding regenerative communication networks, instead of simply eliminating dysfunctional cells, represents a promising framework for developing interventions against sarcopenia and age‐related muscle decline.

## Graphical abstract

Aging transforms the skeletal muscle microenvironment from a balanced, regenerative niche into a maladaptive, degenerative state. This review highlights how disrupted multicellular crosstalk drives chronic inflammation, fibrosis, and neuromuscular dysfunction, ultimately compromising tissue repair and fueling sarcopenia.

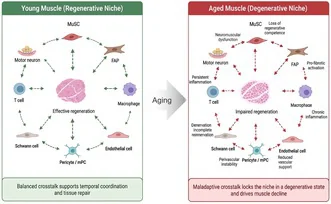

## 1 Introduction

Skeletal muscle is essential for locomotion, metabolic homeostasis, and whole‐body health. With advancing age, skeletal muscle progressively loses mass, strength, and regenerative capacity, ultimately leading to sarcopenia, frailty, disability, and increased mortality. Although muscle aging has traditionally been attributed to intrinsic defects of myofibers or exhaustion of muscle stem cells (MuSCs), accumulating evidence indicates that these mechanisms alone cannot fully explain the progressive decline in muscle function observed during aging (Granic et al. 2023; Grima‐Terren et al. 2024).

Skeletal muscle regeneration is orchestrated by a highly coordinated multicellular niche composed of MuSCs, fibro‐adipogenic progenitors (FAPs), immune cells, vascular cells, Schwann cells, motor neurons, and extracellular matrix (ECM) components. During tissue repair, these cellular populations undergo precisely timed transitions and communicate through cytokines, growth factors, extracellular vesicles (EVs), and direct cell–cell interactions to restore tissue homeostasis (De Micheli et al. 2020; Farup et al. 2015; Sousa et al. 2024). Rather than functioning independently, these populations form an integrated regenerative network in which successful regeneration depends on the temporal and spatial coordination of multiple cell types.

Recent advances in single‐cell RNA sequencing, spatial transcriptomics, multimodal atlases, and lineage‐tracing approaches have fundamentally changed our understanding of skeletal muscle aging. Instead of a simple reduction in stem‐cell number or function, aging is now recognized as a process of population‐level remodeling, characterized by altered cellular composition, aberrant transitional states, disrupted lineage trajectories, and rewired intercellular communication networks (Lai et al. 2024; Moiseeva et al. 2023; Walter et al. 2024). Across the aging muscle niche, MuSCs progressively lose regenerative competence, FAPs become locked in pro‐fibrotic states, immune populations fail to resolve inflammation, vascular support declines, and neuromuscular junctions (NMJs) undergo incomplete reinnervation. These alterations collectively shift the tissue from a regenerative niche toward a degenerative niche.

Importantly, many ligand‐receptor interactions proposed by computational analyses remain correlative, whereas only a limited number of signaling pathways have been functionally validated in vivo. Distinguishing inferred communication networks from causal mechanisms has therefore become a major challenge in translating atlas‐based discoveries into therapeutic strategies for sarcopenia and muscle aging.

In this Review, we summarize recent advances in skeletal muscle aging through the perspective of multicellular communication and population dynamics. We first summarize how aging remodels major cellular populations within the muscle niche, including MuSCs, stromal, immune, vascular, and neuromuscular compartments. We then discuss how disrupted crosstalk among these populations progressively transforms a regenerative microenvironment into a degenerative niche. Finally, we highlight current limitations of atlas‐based studies, distinguish computational predictions from experimentally validated signaling pathways, and discuss emerging therapeutic strategies aimed at restoring multicellular coordination rather than targeting individual cell populations in isolation.

To ensure a balanced and transparent evaluation of the current literature, we conducted a structured search using PubMed and Web of Science databases. The search focused on articles published from 2014 to 2026, utilizing keywords such as “skeletal muscle aging,” “sarcopenia,” “single‐cell RNA sequencing,” “spatial transcriptomics,” and “intercellular communication.” Inclusion criteria prioritized peer‐reviewed original research and high‐resolution multi‐omics atlases investigating cellular population dynamics in in vivo mammalian models (mice and humans). Articles were excluded if they did not mechanistically address multicellular interactions or if they were non‐peer‐reviewed preprints. This methodology was rigorously applied to minimize confirmation bias and objectively integrate findings that both support and challenge current paradigms in muscle aging.

## 2 Population‐Level Remodeling of the Aging Muscle Niche

### 2.1 Myogenic Reserve Remodeling and Regenerative Decline

During skeletal muscle regeneration, MuSCs reside in a quiescent state between the basal lamina and the sarcolemma (Grima‐Terren et al. 2024; Sahinyan et al. 2023). Upon tissue injury, MuSCs are rapidly activated to proliferate as myogenic progenitors, subsequently undergoing differentiation and fusion to reconstruct regenerating myofibers (Sahinyan et al. 2023; Walter et al. 2024). Concurrently, a subset of proliferating cells returns to a quiescent state to replenish the stem cell pool, preserving long‐term regenerative capacity for subsequent injuries (Brett et al. 2020; Sahinyan et al. 2023). Emerging evidence suggests that chronological aging does not entirely abolish this regenerative program but rather disrupts the highly coordinated progression of sequential activation and differentiation steps, thereby compromising regenerative fidelity (Kurland et al. 2023; Walter et al. 2024).

Across various aging animal models, a progressive decline in both the total abundance of MuSCs and the proportion of the subpopulation possessing long‐term self‐renewal capacity has been widely documented (Grima‐Terren et al. 2024; Walter et al. 2024). Beyond numerical depletion, qualitative remodeling of the remaining stem cell population within the niche appears to present a more substantial barrier to regeneration (Lai et al. 2024; Lazure et al. 2023). In geriatric mice aged 20 months or older, a fraction of MuSCs loses the capacity for reversible quiescence and shifts toward a pre‐senescent state (Moiseeva et al. 2023; Walter et al. 2024). This transition is associated with notable impairments in subsequent activation, proliferation, and self‐renewal efficiency (Moiseeva et al. 2023; Walter et al. 2024). Furthermore, time‐resolved trajectory analyses reveal that while aged MuSCs broadly maintain the overall sequence of activation and differentiation, their initial responses are often delayed, and the state transition progresses slowly with highly heterogeneous kinetics compared to young controls (Kurland et al. 2023; Lazure et al. 2023).

Disruptions in metabolic and epigenetic networks appear to contribute directly to this aberrant progression of cellular states (Benjamin et al. 2023; Lazure et al. 2023). Age‐associated depletion of S‐adenosylmethionine (SAM), a critical methyl donor, leads to a reduction in repressive histone methylation (such as H3K9me2/3) and heterochromatin protein 1 (HP1) levels, resulting in the destabilization of heterochromatin architecture within MuSCs (Benjamin et al. 2023; Sahinyan et al. 2023). While this epigenetic instability promotes the accumulation of DNA damage and apoptosis, metabolic interventions aimed at restoring intracellular SAM levels have been shown to re‐establish heterochromatin domains and mitigate age‐related regenerative defects (Benjamin et al. 2023; Wu et al. 2023).

Recent studies further suggest that the decline in regenerative capacity may represent an evolutionary trade‐off to adapt to an adverse aging microenvironment rather than a simple loss of function (Kang et al. 2026). In aged MuSCs, elevated expression of the tumor suppressor NDRG1 strongly suppresses mechanistic target of rapamycin (mTOR) signaling, a principal pathway regulating cellular activation and expansion (Kang et al. 2026). This potential “cellular brake” mechanism prioritizes long‐term cellular survival and resource preservation under chronic inflammatory and oxidative stress at the expense of short‐term tissue repair kinetics (Grima‐Terren et al. 2024; Kang et al. 2026). Pharmacological inhibition of NDRG1 temporarily restores a youthful activation response and accelerates early repair, but repeated injury cycles lead to severe cellular stress and subsequent depletion of the remaining MuSC pool (Kang et al. 2026). These findings illustrate a model of “cellular survivorship bias,” suggesting that the surviving MuSC pool in aged muscle is not optimized for rapid regeneration but is preferentially selected for persistent survival under extreme environmental constraints (Kang et al. 2026).

Notably, independent cross‐species analyses utilizing acute mouse regeneration atlases and chronic human aging datasets demonstrate convergent cellular behaviors (Lai et al. 2024; Walter et al. 2024). Both paradigms demonstrate a notable reduction in the proportion of cells successfully navigating the canonical activation trajectory, accompanied by a shared expansion of aberrant subpopulations exhibiting senescent‐like features or pro‐inflammatory transcriptional signatures (Moiseeva et al. 2023; Walter et al. 2024).

Collectively, age‐related regenerative decline cannot be fully explained by a simple numerical loss of stem cells (Grima‐Terren et al. 2024; Sahinyan et al. 2023). Aged muscle enters the regenerative phase with a compromised and unstable myogenic reserve, leading to a reduced fraction of cells executing productive proliferation and self‐renewal, while populations characterized by stress responses or unresolved transitional states continuously accumulate within the niche (McKellar et al. 2021; Walter et al. 2024). This population‐level remodeling provides a primary cellular foundation for the delayed myofiber repair, incomplete recovery after injury, and progressive loss of regenerative capacity observed during muscle aging (Grima‐Terren et al. 2024; Walter et al. 2024). Beyond the myogenic lineage, age‐associated shifts in cellular composition, key regulators, and functional outcomes across major muscle‐resident populations are summarized in Figure 1.

**FIGURE 1:**
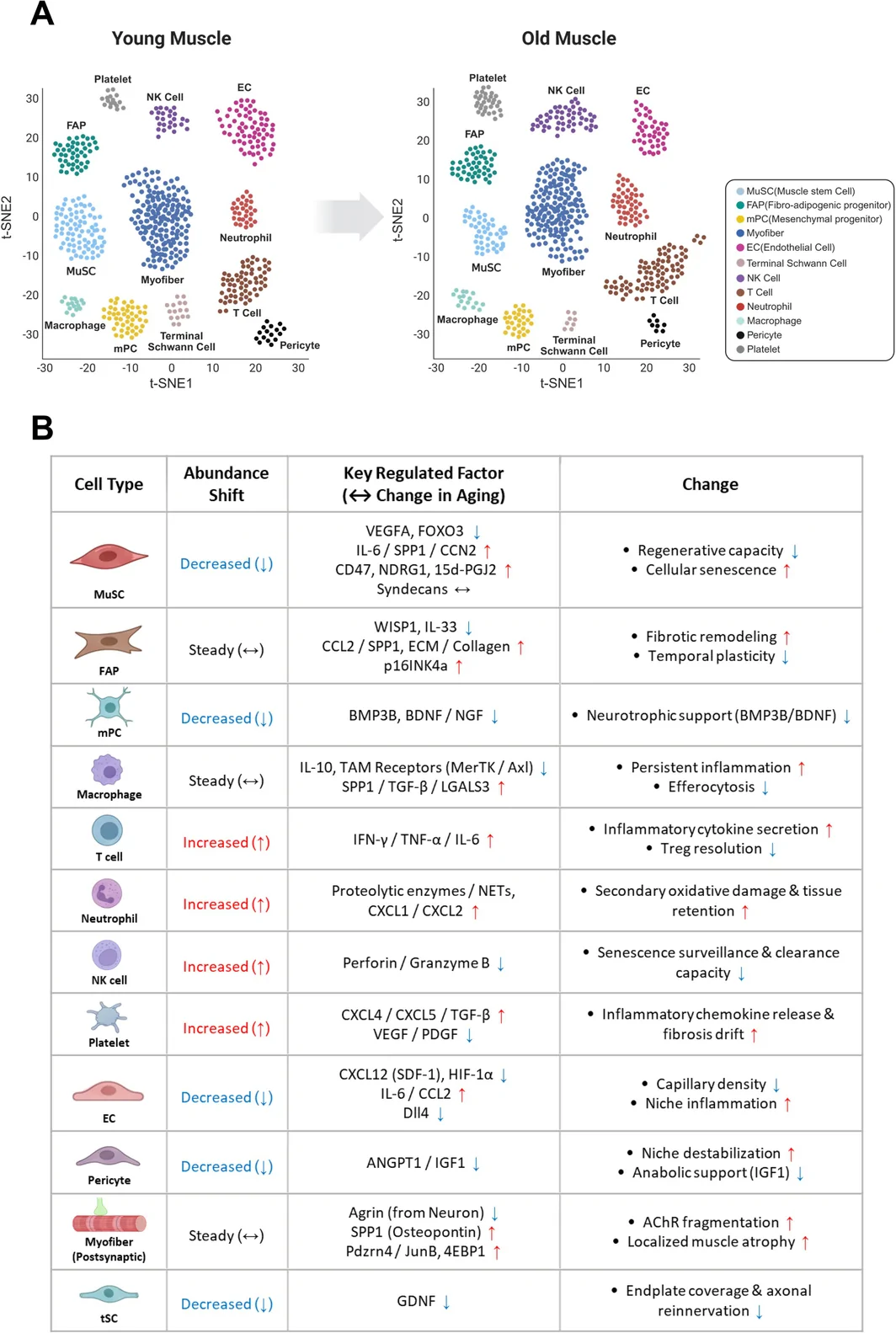
Age‐induced remodeling of the skeletal muscle microenvironment. (A) t‐SNE visualization of single‐cell populations in young (left) and aged (right) skeletal muscle. Note that this representation is designed to highlight the relative age‐dependent shifts in the abundance of each cell type, rather than reflecting their exact absolute in vivo proportions within the whole muscle tissue. (B) A summary table detailing the age‐related changes in cellular abundance alongside alterations in key regulatory factors, encompassing both intracellular signaling molecules and broad expression markers for each cell type.

### 2.2 Immune‐State Persistence and Failed Resolution

Skeletal muscle repair is intrinsically coupled with dynamic shifts within the local immune microenvironment (Sousa et al. 2024; Ziemkiewicz et al. 2021). The initial immune response is postulated to be triggered by platelets recruited upon vascular disruption following tissue injury (Graca, Minden‐Birkenmaier, et al. 2023; Graca, Stephan, et al. 2023). These platelets rapidly degranulate to release chemokines such as CXCL4 and CXCL5, establishing a robust chemoattractant gradient that directs the extravasation of circulating neutrophils and monocytes into the injured niche, thereby initiating early clearance mechanisms (Braganza et al. 2019). This early post‐injury phase is dominated by infiltrating neutrophils and monocytes, which are subsequently superseded by phenotypically diverse macrophage populations that mediate debris clearance and the onset of myogenesis (Ahmadi et al. 2022; Cui et al. 2023; Kawanishi and Machida 2021). As regeneration progresses, the immune compartment must transition from an acute inflammatory state toward resolving and regulatory populations, a prerequisite for the attenuation of stromal remodeling and the re‐establishment of tissue homeostasis (Sousa et al. 2024; Wu et al. 2022).

This spatial and temporal redistribution of immune populations becomes progressively dysregulated in aged skeletal muscle. Both human aging atlases and murine regeneration models demonstrate the persistence of inflammatory, stromal‐associated, and senescence‐related immune programs beyond the optimal window for productive repair (Lai et al. 2024; Sousa et al. 2024). Monocytes and macrophages infiltrating aged tissues circumvent the conventional M1‐M2 dichotomy, instead occupying overlapping, intermediate activation states (Zhang et al. 2024). Notably, rather than stabilizing the tissue, specific aged M2‐like macrophage subsets exhibit a sustained secretion of pro‐fibrotic factors such as TGF‐β and osteopontin (SPP1) (Zhang et al. 2024). This persistent signaling induces adjacent FAPs to deposit excessive collagen, thereby driving fibrotic remodeling (Sousa et al. 2024).

Furthermore, the local accumulation of regulatory T cells (Tregs), which are critical for attenuating acute inflammation, is markedly reduced or delayed in aged muscle (Kuswanto et al. 2016; J. Wu et al. 2022). This defect results in a paucity of pro‐resolving signals, such as IL‐10, which are required for orchestrating macrophage state transitions (Kuswanto et al. 2016; Slaets et al. 2023). In addition, chronic oxidative stress generated by residual neutrophils, combined with pro‐inflammatory cytokines (e.g., IFN‐γ, TNF‐α) secreted by aged T cells, directly impairs the differentiation capacity of MuSCs (Chen et al. 2022; Mittelbrunn and Kroemer 2021; Sousa et al. 2024). Natural killer (NK) cells, which normally execute immune surveillance to clear senescent cells, are also hypothesized to undergo functional alterations during aging (Brauning et al. 2022; Qi and Liu 2023). Such dysfunction may permit the aberrant accumulation of senescent cells within the niche, although further causal validation remains necessary (Larouche et al. 2022).

The consequent regenerative failure is not solely attributable to a quantitative increase in inflammatory factors, but rather to a “temporal mismatch” between the composition of immune states and the sequential repair phases demanded by the tissue (Kurland et al. 2023; Tobin et al. 2021). While MuSCs necessitate an anti‐inflammatory, stable microenvironment to execute myofiber formation (differentiation and fusion) following debris clearance, the aged niche remains replete with early inflammatory signals that propagate tissue disruption and fibrosis (Sousa et al. 2024; Tobin et al. 2021). Consequently, myogenic differentiation and stromal repair are compelled to proceed within an incompatible immune landscape. This immunological desynchronization severely compromises regenerative efficacy, ultimately culminating in incomplete tissue restoration and the formation of persistent fibrotic scars (Grima‐Terren et al. 2024).

### 2.3 FAP State Remodeling and Loss of Stromal Plasticity

Successful skeletal muscle regeneration requires dynamic state transitions within the stromal compartment, particularly by FAPs (De Micheli et al. 2020; Farup et al. 2015). Upon injury, FAPs rapidly accumulate around damaged myofibers and undergo transient expansion (Granic et al. 2023; Negroni et al. 2022). During this phase, FAPs enter a “repair‐supportive state,” secreting ECM components that orchestrate a permissive niche for myogenic progenitor activation and differentiation (Lukjanenko et al. 2016). Following successful myofiber restoration, this expanded FAP population must undergo programmed elimination (apoptosis) or return to a homeostatic quiescent state, thereby facilitating the restoration of normal tissue architecture (Negroni et al. 2022; Porpiglia et al. 2022).

In aged muscle, this cellular plasticity is severely compromised. Recent single‐cell and spatial transcriptomic analyses demonstrate that, during aging, FAPs do not persist as a homogeneous population but instead diverge into a heterogeneous continuum of aberrant transcriptional states linked to inflammation, matrix remodeling, adipogenesis, and senescence (De Micheli et al. 2020). In mouse models, this is characterized by a marked accumulation of *Spp1*‐high (osteopontin), *Ly6a/Sca1* ^+^, and *Dpp4*+ subpopulations, which are closely associated with chronic inflammation and fibrosis (Negroni et al. 2022; Zhang et al. 2024). Analogously, human aging atlases reveal a specific expansion of CD90 (Thy‐1)^+^ FAP subsets that exhibit pro‐fibrotic properties (Farup et al. 2021). Although the molecular markers diverge between species, both models demonstrate that chronological aging precludes the orderly progression of FAP populations through their physiological trajectories (Lai et al. 2024).

It is noteworthy that not all FAPs in aged muscle undergo functional decline. Single‐cell atlases consistently identify preserved FAP subpopulations that maintain anti‐inflammatory and pro‐regenerative gene signatures, continuing to secrete homeostatic ECM components and support myogenesis (Lai et al. 2024). Thus, the fundamental pathology of the aged stromal niche is not an inherent malignant transformation of all stromal cells, but rather the loss of temporal plasticity—the inability of the FAP population to transition fluidly between states and subsequently self‐eliminate upon repair completion (Farup et al. 2021).

The physiological act of transient collagen secretion to stabilize a wound site is a requisite process for successful repair. In aged muscle, however, FAP subsets committed to fibrotic remodeling fail to exit this state, persistently occupying the niche (Zhang et al. 2024). The prolonged retention of these fibrogenic FAP populations precipitates excessive collagen deposition in the endomysium and promotes fibro‐adipose remodeling, where muscle is replaced by fibrotic and adipose tissue, thereby driving the progressive degradation of the entire microenvironment (Granic et al. 2023).

## 3 Intercellular Communication Networks in the Aging Muscle Niche

Rather than numerical or cell‐state alterations alone, it is the disruption of intercellular crosstalk among MuSCs, stromal, immune, and vascular compartments that precipitates the systemic functional decline of skeletal muscle tissue (Farup et al. 2015; Grima‐Terren et al. 2024). This breakdown of multicellular networks represents the cumulative consequence of the population‐level trajectory alterations and aberrant state occupancies detailed in Chapter 2. Within this process, intercellular communication does not operate via isolated, unidirectional pathways, but instead manifests as a highly interconnected multicellular web (De Micheli et al. 2020). To resolve these complex multicellular interactions, it is essential to rigorously distinguish between computationally inferred signaling circuitry derived from omic datasets and causal relationships verified through in vivo functional perturbation and validation experiments (Lai et al. 2024; Lazure et al. 2023). The overarching intercellular communication circuits defining the degenerative niche are schematized in Figure 2, while core secretome alterations across lineages are cataloged in Table 1.

**FIGURE 2:**
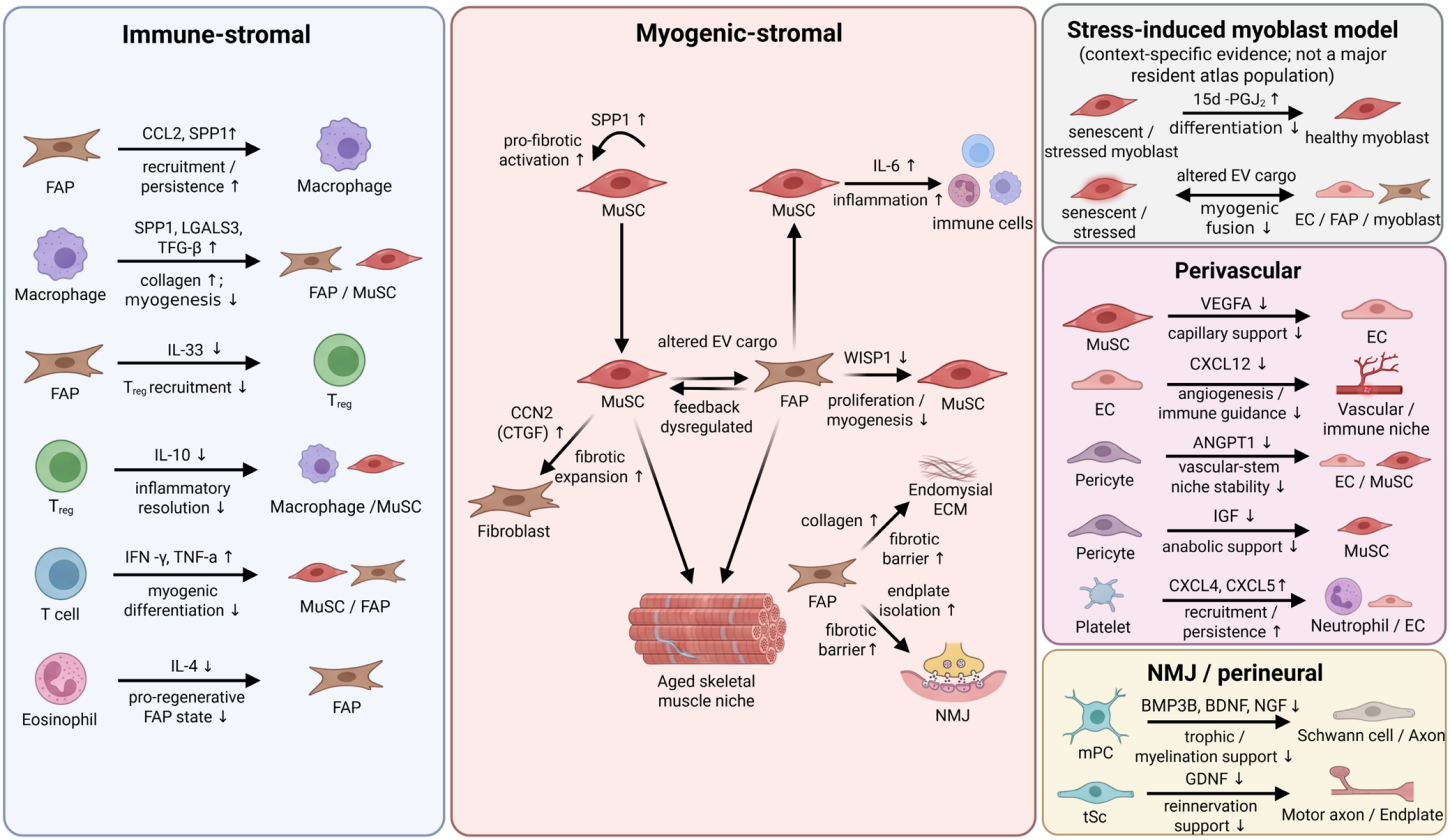
Altered intercellular communication in the aged skeletal muscle niche. This schematic illustrates how aging disrupts signaling networks among resident cells, driving the transition toward a degenerative microenvironment across the immune‐stromal, myogenic‐stromal, perivascular, and neuromuscular (NMJ/perineural) compartments. Within the diagram, straight arrows (→) indicate paracrine signaling from a source cell to a target cell, whereas curved (↶) and bidirectional (⇄) arrows represent autocrine signaling and bidirectional communication, respectively. Upward (↑) and downward (↓) arrows denote age‐associated increases or decreases in the secretion of the indicated factors, along with their subsequent functional impacts and physiological changes induced in the recipient cells. Additionally, the top right panel depicts senescence‐associated bystander effects based on stress‐induced myoblast models.

**TABLE 1:** Core secretome alterations and multicellular crosstalk in the aging skeletal muscle niche.

Lineage	Cell type	Secreted factor	Age‐related shift	Target cell	Hedged pathological/Physiological impact
Myogenic	MuSC	VEGFA	↓	Endothelial cells	Regulates spatial proximity to capillaries and preserves the quiescent stem cell state
		IL‐6	↑	Immune & stromal	Promotes chronic low‐grade inflammation and recruits immune cells in the aging niche
		SPP1 (Osteopontin)	↑	Macrophages & self	Drives pro‐fibrotic macrophage activation and autocrine niche degeneration
		CCN2 (CTGF)	↑	FAPs & fibroblasts	Stimulates FAPs to drive aberrant collagen synthesis and tissue fibrosis
	Myoblast	15d‐PGJ2	↑	Healthy myoblasts	Propagates cellular senescence and arrests myogenic differentiation via covalent modification of HRas (Cys184)
		EVs	↔	FAPs & myoblasts	Delivers senescent SASP cargo to FAPs and impairs reciprocal myogenic fusion
Stromal/Mesenchymal	FAP	WISP1	↓	MuSCs	Activates AKT signaling in MuSCs to promote productive proliferation and lineage progression
		IL‐33	↓	Tregs	Recruits anti‐inflammatory Tregs to resolve acute post‐injury inflammatory responses
		CCL2	↑	Macrophages	Sustains macrophage recruitment and delays phenotypic switching toward resolution
		SPP1 (Osteopontin)	↑	Macrophages	Reinforces the pathogenic macrophage‐FAP feedback loop to drive chronic fibrosis
		ECM (Collagen)	↑	Niche environment	Forms a physical scar barrier that isolates NMJs and impairs muscle fiber repair
		EVs	↔	MuSCs & macrophages	Spreads paracrine senescence and reinforces pro‐fibrotic signaling in the stroma
	mPC	BMP3B	↓	Schwann cells	Maintains skeletal muscle tissue integrity and counteracts sarcopenic muscle wasting
		BDNF	↓	Schwann cells/Axons	Provides essential neurotrophic protection for synaptic apparatus and NMJ stability
		NGF	↓	Schwann cells/Axons	Promotes synaptic structural maintenance and axonal guidance during reinnervation
Immune	Marcrophagy	IL‐10	↓	FAPs & MuSCs	Drives myeloid cell switching toward resolution and initiates myogenic repair
		SPP1 (Osteopontin)	↑	FAPs	Stimulates FAP survival and activates pro‐fibrotic and adipogenic differentiation pathways
		LGALS3 (Galectin‐3)	↑	FAPs	Promotes fibro‐adipose matrix remodeling in cooperation with chronic TGF‐b signals
		TGF‐β	↑	FAPs & MuSCs	Upregulates extracellular matrix synthesis and blocks functional myofiber fusion
	Treg	IL‐10	↓	Macrophages/MuSCs	Drives myeloid cell switching from pro‐inflammatory to pro‐regenerative states
	T cell	IFN‐γ	↑	MuSCs & myoblasts	Delays MuSC activation kinetics and arrests early myogenic differentiation
		TNF‐α	↑	MuSCs & FAPs	Blocks myogenic differentiation and exacerbates chronic inflammatory muscle wasting
	Eosinophil	IL‐4	↓	FAPs	Induces transient FAP activation to support early myogenesis and prevent adipogenic drift
	Platelet	CXCL4	↑	Neutrophils/ECs	Guides early neutrophil recruitment; chronic activation prolongs acute tissue damage
		CXCL5	↑	Neutrophils/ECs	Regulates vascular‐immune homeostasis and immune infiltration dynamics during early repair
Vascular	EC	CXCL12	↓	Neutrophils & ECs	Coordinates capillary‐niche remodeling and guides spatial immune recruitment
	Pericyte	ANGPT1	↓	MuSCs & ECs	Stabilizes endothelial‐niche junctions and promotes MuSC return to quiescence
		IGF1	↓	MuSCs & progenitors	Upregulates anabolic signaling for myofiber hypertrophy and structural repair
Glial/NMJ	tSC	GDNF	↓	Motor neurons	Protects denervated endplates and maintains synaptic stability to facilitate reinnervation

*Note:* The table summarizes key secreted mediators, their age‐related shifts, and their direct intercellular impacts across major functional cell lineages (myogenic, stromal, immune, vascular, and glial/neuromuscular) during skeletal muscle aging and regeneration. Arrows and symbols indicate the direction of age‐related alterations within the microenvironment: (↑) increased expression or pathological hyper‐activation; (↓) decreased expression, impaired recruitment, or localized depletion; and (↔) altered molecular composition or dysregulated cargo.

Abbreviations: EC, endothelial cell; EVs, extracellular vesicles; FAP, fibro‐adipogenic progenitor; mPC, mesenchymal progenitor; MuSC, muscle stem cell; NMJ, neuromuscular junction; tSC, terminal Schwann cell.

### 3.1 MuSC‐FAP Crosstalk Regulates the Timing of Tissue Repair

The dynamic shifts in cellular populations described above ultimately culminate in the dysregulation of refined intercellular communication networks. Efficient muscle repair fundamentally relies on precise temporal coordination between the myogenic and stromal compartments, a homeostatic relationship that becomes progressively desynchronized with chronological aging (Kurland et al. 2023; Walter et al. 2024). Immediately following injury, FAPs undergo transient expansion to establish a supportive, permissive microenvironment that facilitates productive MuSC activation, proliferation, and differentiation (Farup et al. 2015). This stromal support is governed not only by soluble paracrine factors, but also by the biophysical composition and mechanical properties of the ECM (Chazaud 2020; Rossman et al. 2017). For instance, FAP‐derived WISP1 promotes MuSC proliferation and myogenesis by activating downstream integrin‐AKT signaling pathways (Lukjanenko et al. 2019). Concurrently, fibronectin within the stem cell niche interacts with the integrin‐PTK2/FAK axis to regulate cellular adhesion, polarity, and niche‐derived signal transduction (Lukjanenko et al. 2016). Additional niche modulators, including THBS1‐CD47 signaling, may further modulate MuSC expansion and regenerative responses depending on the microenvironmental context (Porpiglia et al. 2022).

Communication between MuSCs and FAPs is inherently reciprocal, shifting dynamically across sequential phases of regeneration. Once FAPs establish the initial myogenesis‐permissive niche, regenerating myogenic cells provide feedback to facilitate the return of the stromal compartment to homeostatic quiescence (McKellar et al. 2021). While FAP‐derived EVs enhance early myogenesis, EVs secreted by MuSCs and myogenic progenitors during later stages serve to restrict excessive FAP expansion and fibro‐adipogenic differentiation (De Micheli et al. 2020). This precisely timed signaling exchange is best understood within the broader context of stage‐dependent ligand responsiveness. Indeed, cycling myogenic progenitors dynamically alter the expression patterns of surface syndecans—heparan sulfate proteoglycans (HSPGs) that act as co‐receptors—to concentrate and present niche ligands, such as FGF2, TGF‐β1, and RSPO3, to their primary receptors (De Micheli et al. 2020; McKellar et al. 2021). This mechanism enables progenitors to selectively amplify and process specific cues required for their immediate cellular state. Although these ligand‐responsiveness profiles alone do not definitively map unidirectional regulation, they suggest that myogenic cells are not merely passive recipients of stromal support, but rather actively sense and interpret complex microenvironmental inputs.

This highly coordinated interactive program is prone to pathological alteration during aging. A reduction in biophysical and biochemical support, characterized by the decline of FAP‐derived WISP1 and the depletion of fibronectin derived from multiple resident populations (including MuSCs and immune cells), imposes an increased extrinsic barrier to MuSC‐mediated regeneration (Lukjanenko et al. 2016, 2019). Concurrently, epigenetically perturbed aged MuSCs exert an aberrant secretome, releasing factors such as IL‐6 and SPP1 that alter adjacent FAP behavior and potentially stimulate excessive FAP expansion and fibrogenic remodeling within the senescent niche (Moiseeva et al. 2023; Zhang et al. 2024). Although historical ex vivo culture constraints restricted comprehensive secretome profiling due to the rapid loss of MuSC quiescence, recent investigations demonstrate that aged MuSCs actively modify their microenvironment through distinct paracrine signaling cascades (Moiseeva et al. 2023).

Consequently, the functional decline of MuSCs does more than weaken myofiber formation; it progressively drives a degenerative shift in the stromal environment that further impedes tissue repair. However, robust experimental support for these proposed signaling axes remains somewhat limited. While the downregulation of FAP‐derived WISP1 and the pathogenic roles of MuSC‐derived IL‐6 and SPP1 have been functionally validated in murine models, the chronic age‐related alterations in EV‐mediated feedback loops and the vast majority of multi‐omic ligand‐receptor predictions remain to be definitively elucidated (Moiseeva et al. 2023; Zhang et al. 2024). Future investigations must therefore delineate the precise temporal sequences, spatial localization, and cell‐specific contributions of MuSC‐FAP signaling to discover targeted interventions that restore homeostatic communication without indiscriminately suppressing supportive FAP states.

### 3.2 Immune–Stromal Crosstalk Coordinates Inflammatory Resolution

Chronic fibrosis in skeletal muscle is not merely a consequence of the absolute intensity of the initial inflammatory response. The fundamental pathology lies in “failed resolution”—the inability to timely terminate pro‐inflammatory repair programs before stromal remodeling becomes permanently entrenched (Sousa et al. 2024; Tobin et al. 2021). Central to this failure is a persistent pathogenic feedback loop established between macrophages and stromal cells (Braganza et al. 2019; Zhang et al. 2024).

The homeostatic immune response to injury is intrinsically subjected to strict sequential regulation. During the initial phase, macrophage‐mediated efferocytosis of apoptotic cells and tissue debris acts as a critical molecular switch transitioning the tissue from acute inflammation to regenerative repair; in this process, the TAM receptor pathway, including macrophage surface MerTK and Axl, plays an indispensable role (Cui et al. 2023; Duong et al. 2021). Concurrently, localized stromal cues finely orchestrate this state transition. FAP‐like stromal populations secrete IL‐33 to induce the local accumulation of regulatory T cells (Tregs); indeed, exogenous IL‐33 administration in aged mice effectively restores both Treg accumulation and regenerative capacity (Kuswanto et al. 2016; Wu et al. 2022). Subsequently, anti‐inflammatory cytokines such as IL‐10, secreted by these recruited Tregs and resolving macrophages, attenuate acute inflammation and facilitate functional niche recovery (Sahinyan et al. 2023). Type 2 immune signaling also participates in this restorative phase, where eosinophil‐derived IL‐4 can induce the pro‐regenerative activation of FAPs (Slaets et al. 2023). However, recent loss‐of‐function experiments utilizing knockout (KO) models have revealed that these Type 2 immune pathways may not be strictly required for regeneration in specific injury paradigms, suggesting that researchers must refrain from prematurely dichotomizing specific immune pathways into simplistic “pro‐regenerative” or “pro‐fibrotic” categories (Sousa et al. 2024; Ziemkiewicz et al. 2021).

Pathological consequences emerge when the activation of this immune resolution switch is delayed. Under these conditions, cellular communication signals originally tasked with coordinating repair diverge into a self‐reinforcing circuit that accelerates mutual pathology. According to single‐cell and spatial transcriptomic analyses, aged FAPs secrete substantial amounts of CCL2 and SPP1 (osteopontin) into the extracellular space, which entraps macrophages in a pro‐inflammatory state and continuously recruits them to the injury site (Moiseeva et al. 2023; Zhang et al. 2024). This stimulated macrophage population, in turn, highly expresses SPP1, LGALS3 (Galectin‐3), and TGF‐β, thereby compelling adjacent FAPs to deposit excessive collagen‐rich ECM (Ahmadi et al. 2022; Zhang et al. 2024). Thus, rather than functioning unidirectionally, immune and stromal cells engage in an unrelenting pathogenic relay that translates unresolved inflammation into irreversible fibrosis. Simultaneously, chronic exposure to pro‐inflammatory cytokines (e.g., TNF‐α, IFN‐γ) persistently released by these immune cells directly compromises the differentiation capacity of MuSCs, thereby suppressing tissue regeneration (Dumke and Lees 2011; Xu et al. 2024).

Consequently, this bidirectional crosstalk emphasizes that the critical determinant of tissue pathology is not the static phenotypic classification of immune populations, but rather the precise temporal regulation of signaling circuitry (Sousa et al. 2024; Thomson 2018). Macrophage signaling pathways that are initially indispensable for early debris clearance can transition into maladaptive, self‐reinforcing programs that sustain stromal fibrosis during later repair stages. The primary pathological driver within the aging niche, therefore, is whether the macrophage‐FAP communication circuit is appropriately extinguished following repair completion, or whether it surpasses its physiological timeframe to continuously mediate microenvironmental signal disruption.

To date, the majority of functional evidence regarding these immune‐stromal checkpoints has been derived from acute injury or muscular dystrophy models. The specific temporal hierarchies governing CCL2, SPP1, LGALS3, and TGF‐β circuits during physiological chronic aging remain to be fully elucidated. Because ligand‐receptor predictions derived solely from single‐cell data cannot definitively establish causality, future investigations must integrate age‐specific spatial transcriptomic profiling with time‐controlled perturbation experiments targeting specific cell lineages or temporal windows. Such approaches are essential to identify precise strategies for restoring immune resolution.

### 3.3 Vascular–Regenerative Crosstalk Shapes the Repair Microenvironment

The regenerative capacity of skeletal muscle is substantially governed by the capillary‐adjacent microenvironment. In youthful tissue, MuSC‐derived VEGFA induces the proliferation and migration of local endothelial cells (ECs), thereby maintaining close spatial proximity between the stem cell pool and the vascular network (Dobner et al. 2024; Jiang et al. 2024). This physical juxtaposition facilitates direct engagement between EC‐expressed Dll4 and Notch receptors on MuSCs, a signaling axis required to suppress aberrant activation and sustain a stable quiescent state (Dobner et al. 2024; Farup et al. 2015). Pericytes enveloping the capillary endothelium exert precise regulatory control over this niche, wherein pericyte‐derived angiopoietin‐1 (ANGPT1)‐TIE2 signaling promotes the reversion of activated myogenic progenitors back to a quiescent state to sustain self‐renewal (Grima‐Terren et al. 2024; Jiang et al. 2024). Consequently, ECs and mural cells extend beyond their classical role in perfusion to function as active signaling nodes that directly modulate myogenic state transitions.

Age‐associated declines in capillary integrity, EC function, and perivascular architecture compromise this regulatory system, ultimately impairing vascular‐dependent regenerative responses. For instance, age‐related deficits in hypoxia‐inducible factor 1‐alpha (HIF‐1α) signaling attenuate the capacity of both ECs and myogenic cells to sense and mount appropriate responses to localized hypoxic stress during the early phases of injury (Dobner et al. 2024; Ron‐Harel et al. 2018). This blunted hypoxic response diminishes compensatory angiogenesis, potentially precipitating the collapse of the capillary networks essential for sustained MuSC activity (Wu et al. 2023). However, definitive causal evidence demonstrating that specific vascular defects directly drive aberrant MuSC behavior and functional decline during physiological aging alone remains limited. Nevertheless, the observation that ablating CXCL12—a pro‐angiogenic factor primarily secreted by ECs and stromal cells—in severe injury models results in severely impaired angiogenesis, persistent immune infiltration, and fibro‐adipose remodeling illustrates the mechanistic principle that the disruption of vascular‐derived cues can propagate pan‐niche pathology (Dobner et al. 2024; Sousa et al. 2024).

Vascular signals also exert direct regulatory effects on the mature myofiber compartment. Under conditions of disuse atrophy or diabetes, EC‐derived Dll4 activates Notch2 receptors within adjacent myofibers to induce atrophy, and pharmacological inhibition of this pathway significantly mitigates muscle loss in murine models (Dobner et al. 2024; Grima‐Terren et al. 2024). In stark contrast to its role in maintaining MuSC quiescence, this finding represents a classic paradigm of pleiotropy, wherein an identical vascular‐derived ligand (Dll4) dictates diametrically opposed biological outcomes—tissue preservation versus degradation—depending on the specific receiving cell (Dobner et al. 2024). Although these disease‐model mechanisms cannot be directly extrapolated to physiological aging, they firmly establish that the biological consequences of vascular signals are highly contingent upon target cell identity and the prevailing microenvironmental context.

### 3.4 Senescence‐Associated Communication Amplifies Repair Failure

In aged skeletal muscle, the resolution phase of repair is characterized by the persistent retention of senescent MuSCs, FAPs, and macrophages, which physiological repair mechanisms fail to eliminate (Englund et al. 2021; Moiseeva et al. 2023). Because conventional senescence markers—such as p16^INK4a^, p21^CIP1^, SA‐β‐gal activity, and DNA damage response indicators—capture overlapping yet distinct cellular states, this persistence is best defined as a composite cellular program rather than a single molecular identity. Once retained within the niche, these cells are not merely growth‐arrested; they actively establish a pro‐inflammatory microenvironment that induces secondary senescence in adjacent cells, thereby repressing MuSC proliferation. Empirical evidence demonstrates that targeted ablation of senescent cells or pharmacological attenuation of the senescence‐associated secretory phenotype (SASP) accelerates regeneration, limits fibrotic remodeling, and preserves contractile force across age groups, whereas the ectopic transplantation of senescent cells exacerbates regenerative delays (Englund et al. 2021; Moiseeva et al. 2023).

The MuSC‐stromal axis exemplifies how cellular senescence subverts homeostatic niche interactions into maladaptive cross‐regulatory pathways. CCN2, also known as connective tissue growth factor (CTGF), is markedly upregulated in the secretome of senescent MuSCs (Englund et al. 2021; Moiseeva et al. 2023). Forced overexpression of CCN2 in MuSCs compromises their regenerative engraftment potential and stimulates local fibroblast expansion, thereby exacerbating fibrotic deposition. Conversely, the genetic knockdown of CCN2 in aged MuSCs significantly improves regenerative outcomes and attenuates fibrotic remodeling (Englund et al. 2021; Moiseeva et al. 2023). These findings indicate that aged MuSCs do not simply experience a passive loss of responsiveness to niche support; rather, they function as active instigators that propagate their intrinsic defects via paracrine signaling, redirecting adjacent stromal cells toward pathogenic fibroblast expansion and matrix accumulation.

Furthermore, FAP senescence constitutes a parallel mechanism through which regenerative failure propagates across the immune‐stromal network. The chronic macrophage‐FAP feedback loop delineated in Section 3.2 is mechanistically sustained by specific SASP factors, notably CCL2 and SPP1, which are secreted as FAPs transition into a senescent state (Moiseeva et al. 2023; Zhang et al. 2024). In vitro assays confirm that the conditioned medium from primary stress‐induced senescent FAPs strongly promotes macrophage chemotaxis and skews their polarization toward an M2‐like phenotype (Zhang et al. 2024). Consequently, the elevated secretion of CCL2 and SPP1 transcends standard senescence markers, serving as critical paracrine mediators that sequester resolving macrophages within the lesion and prolong matrix‐remodeling signals. In progeroid and dystrophic mouse models, senescent FAPs directly impair the functional capacity of myogenic progenitors, whereas the targeted elimination of senescent FAPs and macrophages via fisetin‐mediated senolysis restores the MuSC pool and regenerative efficacy (Englund et al. 2021; Zhang et al. 2024). However, whether senescent FAPs initiate this pathological cascade during physiological aging or whether they emerge downstream of cumulative chronic damage remains to be elucidated.

Senescence‐associated communication extends beyond the immediate local circuitry to exert broader tissue‐level effects. Stress‐induced prematurely senescent human myoblasts release an increased abundance of small EVs, which penetrate adjacent capillary ECs to induce senescence‐associated phenotypes and compromise vascular functionality (Englund et al. 2021; Grima‐Terren et al. 2024). This mechanism provides a plausible molecular link wherein myogenic stress precipitates the deterioration of the vascular networks required for effective repair. Additionally, doxorubicin‐induced senescent myoblasts secrete 15‐deoxy‐Δ12,14‐prostaglandin J2 (15d‐PGJ2), a bioactive lipid that potently suppresses the proliferation and fusion of neighboring healthy myoblasts (Englund et al. 2021). Mechanistic tracking within the recipient normal myoblasts revealed that 15d‐PGJ2 covalently and irreversibly modifies the Cys184 residue of the HRas protein, leading to aberrant ERK pathway hyperactivation and a subsequent blockade of myogenic differentiation (Grima‐Terren et al. 2024). Although these EV‐ and lipid‐mediated mechanisms of secondary senescence and the bystander effect have been primarily characterized in severe stress‐induced models, they provide compelling evidence that the SASP repertoire has evolved beyond soluble cytokines to encompass transferable, nonprotein signaling modalities.

The fundamental question of why these deleterious senescent populations evade timely clearance and persist within aged muscle remains a critical unresolved challenge (Nishikawa et al. 2021). In other tissues, senescent cells express NKG2D ligands (e.g., MICA and ULBP2) to elicit recognition and subsequent elimination by NK cells (Brauning et al. 2022; Qi and Liu 2023). However, whether a homologous failure in precise immune surveillance permits the persistent survival of senescent cells in aged skeletal muscle has yet to be directly established. Collectively, current evidence indicates that unresolved cellular senescence propagates cascading dysfunction—from MuSCs to stromal cells, from FAPs to the immune compartment, and from stressed myoblasts to the adjacent vasculature. The retention of senescent cells, therefore, transcends a simple cell‐autonomous growth arrest; by prolonging pathogenic signaling throughout the microenvironment, this disrupted communication network functions as a primary amplifier of regenerative failure in aged skeletal muscle.

## 4 Integration of the Neuromuscular Axis and the Muscle Niche

The neuromuscular axis represents a specialized microdomain where multicellular interactions within skeletal muscle are spatially concentrated at the synaptic junction. Within this niche, structural alterations of the motor unit, the adaptability of terminal Schwann cells (tSCs), stromal cell support, and localized MuSC responses converge to dictate whether a denervated endplate undergoes successful reinnervation or permanent disconnection. The motor nerve terminals, postsynaptic myofiber domains, tSCs, and perisynaptic stromal cells cooperatively interact at the NMJ to maintain synaptic stability and modulate muscle function. Notably, synaptic destabilization and motor unit remodeling often precede detectable sarcopenia, fundamentally modulating the regenerative and metabolic landscape of the muscle (Granic et al. 2023; Grima‐Terren et al. 2024). Furthermore, this intercellular communication is highly reciprocal; partial denervation alters myofiber‐derived secretomes, MuSC behaviors, and stromal states, whereas a loss of stromal support, chronic inflammation, or impaired glial assistance conversely compromises synaptic maintenance and reinnervation (Farup et al. 2015; Fuertes‐Alvarez and Izeta 2021). Consequently, the NMJ represents a spatially specialized microdomain that warrants dedicated investigation within the study of the aging muscle microenvironment.

### 4.1 Motor Unit Remodeling, Denervation, and Postsynaptic Instability

Age‐associated decline in neuromuscular function does not occur uniformly across the tissue. Instead, it involves a progressive loss of motor neurons, incomplete collateral sprouting, aberrant reinnervation, and the preferential denervation of fast‐twitch glycolytic motor units, which collectively culminate in a mosaic pattern of denervated, reinnervated, and structurally unstable myofibers (Gonzalez‐Freire et al. 2014; Granic et al. 2023). This mosaic remodeling contributes to functional muscle weakness prior to significant muscle mass loss. In aged mice, even in the absence of severe global denervation, the proportion of MuSCs localizing to the postsynaptic myonuclei to facilitate repair is notably reduced (Campanario et al. 2026; Lai et al. 2024). Conversely, experimental depletion of MuSCs accelerates NMJ deterioration and exacerbates muscle weakness (Campanario et al. 2026; Gonzalez‐Freire et al. 2014). Although a subset of MuSCs accumulates near compromised NMJs under neuromuscular stress, this protective response is compromised in aged or antioxidant‐deficient (e.g., Sod1‐deficient) muscles, suggesting that MuSCs act as localized niche helpers supporting synaptic maintenance (Gonzalez‐Freire et al. 2014).

To examine the molecular underpinnings of progressive neuromuscular decline under physiological conditions, researchers frequently employ experimental denervation models, such as sciatic nerve transection. Denervation profoundly remodels the signaling network between myofibers and stromal cells. Denervated myofibers exhibit elevated secretion of SPP1 (osteopontin) and TGF‐β1, which induces premature MuSC activation characterized by JunB upregulation; in the context of concurrent muscle injury, this compromised signaling network impairs myogenic differentiation and myofiber maturation (Farup et al. 2015; Gonzalez‐Freire et al. 2014). Moreover, denervation serves as a potent driver of aberrant FAP accumulation (Zhang et al. 2024). Despite the absence of the typical macrophage or MuSC expansion observed during standard wound healing, denervated FAPs exhibit persistent STAT3 pathway activation and excessive IL‐6 secretion, which directly promotes chronic muscle atrophy and fibrotic scarring (Farup et al. 2015; Zhang et al. 2024). Thus, age‐related loss of neural input may initiate a pathogenic cycle where stromal‐induced fibrotic barriers physically obstruct axonal reentry, further isolating the NMJ. However, whether this self‐reinforcing pathogenic cascade occurs under the gradual, slow cycles of denervation and reinnervation characteristic of human aging remains to be fully elucidated.

As signal transmission at the synaptic cleft destabilizes with age, it precipitates postsynaptic acetylcholine receptor (AChR) fragmentation—a hallmark of NMJ aging—accompanied by reduced synaptic contact area and partial denervation (Gonzalez‐Freire et al. 2014; Mejia Maza et al. 2021). Physiologically, the agrin‐LRP4‐MuSK signaling axis clusters receptors and maintains the structural integrity of the motor endplate, but the efficacy of this pathway declines in the aged synapse (Gonzalez‐Freire et al. 2014; Wu et al. 2012). To dissect the individual contributions of electrical activity and biochemical cues to synaptic transcription, prior studies have compared nerve transection, blockade of acetylcholine release, and muscle‐specific MuSK deletion models (Ang et al. 2022; Wu et al. 2012). While the loss of electrical stimulation triggers canonical denervation responses, such as ectopic Chrng expression, MuSK deficiency alters a distinct set of synaptic genes (Wu et al. 2012). Specifically, muscle‐specific deletion of Pdzrn4, a Golgi‐associated protein identified in these workflows, induces physical NMJ fragmentation, confirming that postsynaptic transcriptional dysregulation directly compromises synaptic structure (Ang et al. 2022; Campanario et al. 2026).

Nevertheless, structural fragmentation does not invariably correlate with functional synaptic failure. For instance, muscle‐specific activation of 4EBP1 leads to morphological fragmentation of the receptor apparatus, yet it accelerates receptor turnover, stimulates MuSC‐mediated recruitment of new postsynaptic myonuclei, and ultimately enhances synaptic transmission efficiency (Ang et al. 2022; Campanario et al. 2026). Therefore, evaluating the progressive aging of the NMJ requires a comprehensive analysis integrating structural, electrophysiological, and transcriptional parameters, and the active preservation of the postsynaptic domain via the 4EBP1‐mTORC1 axis represents a potential therapeutic target to rejuvenate NMJ architecture in sarcopenia.

### 4.2 Terminal Schwann Cell Plasticity and Reinnervation Failure

Terminal Schwann cells serve as integral cellular mediators orchestrating the processes of denervation and subsequent reinnervation (Afzali et al. 2018; Fuertes‐Alvarez and Izeta 2021). Upon nerve terminal disconnection, tSCs extend extensive processes to guide regenerating axons, ensuring they accurately reinnervate the original motor endplate. Indeed, the experimental ablation of tSCs in adult murine models precipitates severe endplate fragmentation and fundamentally impairs both NMJ reinnervation and the recovery of muscle contractile force (Campanario et al. 2026; Gonzalez‐Freire et al. 2014). This restorative response is not uniform but highly dynamic; during the remodeling phase, a specialized Schwann cell subpopulation, termed ‘tSC‐B’, exhibits localized expansion to preferentially support cell migration, polarization, and morphogenesis (Campanario et al. 2026). Within this context, SPP1 (osteopontin) emerges as a critical molecular conduit that mediates crosstalk among resident Schwann cells, recruited phagocytes, and tSCs to facilitate Schwann cell proliferation and subsequent reinnervation (Campanario et al. 2026; Zhang et al. 2024). Neutralization of SPP1 in skeletal muscle results in a marked reduction in tSC abundance and spatial distribution, leading to the accumulation of denervated endplates (Campanario et al. 2026). Consequently, while direct muscle stimulation maintains contractile force, nerve‐evoked force generation is significantly attenuated, suggesting an arrest in tSC maturation prior to full glial differentiation. This phenomenon illustrates a classic paradigm of context‐dependent plasticity: whereas SPP1 drives detrimental chronic fibrosis within the broader stromal microenvironment, it simultaneously acts as an indispensable neurotrophic factor within the NMJ niche to promote synaptic restoration.

However, direct evidence confirming that this specific mechanism operates identically during physiological aging remains relatively sparse. In aged mouse models deficient in the nerve growth factor (NGF) receptor p75NTR, studies report a reduction in tSC endplate coverage, exacerbated denervation, and a concurrent decline in AChR stability and muscle strength (Fuertes‐Alvarez and Izeta 2021). Although the systemic nature of the p75NTR‐knockout model precludes definitive attribution solely to tSC‐intrinsic defects, these findings suggest that p75NTR signaling may play a crucial role in maintaining NMJ resilience during aging. The extent to which analogous age‐related alterations in tSC trajectories occur within the human muscle microenvironment awaits further direct empirical validation.

### 4.3 Stromal Niche Support and NMJ Disconnection

Stromal cell populations within skeletal muscle provide an additional localized supportive layer essential for the maintenance of neuromuscular architecture. Mesenchymal progenitors expressing PDGFRα, including FAP‐like cells, preferentially reside in close spatial proximity to motor axons and tSCs at the NMJ (Negroni et al. 2022; Uezumi et al. 2021). The experimental depletion of these stromal populations precipitates an accumulation of partially or fully denervated endplates and induces the structural disorganization of Schwann cells, implicating these mesenchymal cells as direct contributors to NMJ structural stability (Campanario et al. 2026; Uezumi et al. 2021).

A prominent molecular link connecting mesenchymal progenitor aging to NMJ degeneration is the protein BMP3B. While Bmp3b is abundantly expressed by intramuscular mesenchymal progenitors, its expression levels exhibit a significant, age‐dependent decline in both mice and humans (Uezumi et al. 2021). In vitro, BMP3B is sufficient to support Schwann cell identity and neural myelination, and the sustained expression of Bmp3b in the mesenchymal cells of aged mice effectively attenuates NMJ degeneration, muscle atrophy, and global functional decline compared to controls (Uezumi et al. 2021). Thus, stromal aging not only promotes fibrotic remodeling but also induces the depletion of localized trophic support, thereby compromising neuromuscular stability.

Further insights from peripheral nerve injury models suggest that the efficacy of reparative signaling between Schwann cells and mesenchymal cells may progressively deteriorate with age. Following nerve injury, CD90+ mesenchymal cells are known to upregulate NGF in the vicinity of p75NTR‐expressing Schwann cells, initiating a localized neurotrophic response to denervation (Campanario et al. 2026; Farup et al. 2021). Additionally, a specific FAP subpopulation expressing Ret and Gfra1 can sense Schwann cell‐derived GDNF to subsequently secrete brain‐derived neurotrophic factor (BDNF) (Campanario et al. 2026). The specific genetic ablation of Bdnf in FAPs delays remyelination, and notably, the injury‐induced induction of Bdnf is markedly blunted in aged FAPs (Campanario et al. 2026). Although these studies do not exclusively model physiological NMJ aging, they provide highly plausible molecular pathways through which stromal senescence could obstruct successful reinnervation.

## 5 Conclusions and Future Perspectives

This Review highlights the shift from a traditional, myofiber‐focused view of age‐related regenerative decline toward a broader, multicellular model of skeletal muscle aging. Muscle aging is closely associated with altered population‐level dynamics and disrupted lineage transitions (McKellar et al. 2021; Walter et al. 2024). Within this degenerative niche, MuSCs adopt a ‘cellular survivorship bias’—prioritizing survival under chronic stress over immediate regenerative activation (Kang et al. 2026). Concurrently, senescent FAPs interact with non‐resolving M2‐like macrophages to create a pro‐fibrotic loop, gradually transforming a coordinated regenerative microenvironment into a degenerative niche (Moiseeva et al. 2023).

As multi‐omics datasets continue to expand, a key challenge is distinguishing molecular correlations from in vivo causality (Farup et al. 2021; Iyer et al. 2021). While computational predictions provide valuable maps of intercellular crosstalk, functional validation through targeted approaches—such as CD47 blockade, WISP1 supplementation, or senescent FAP clearance—remains essential to identify causal regulatory nodes and translate observational findings into potential therapies.

Furthermore, addressing the species‐specific translational gap between acute, highly synchronized murine injury models and the chronic, insidious muscle attrition characteristic of human sarcopenia remains an imperative challenge. Moving forward, integrating rodent regeneration dynamics with longitudinal human datasets under basal, uninjured conditions will be indispensable for the design of clinically viable therapeutic interventions.

Ultimately, effective rejuvenation strategies should adopt a multi‐targeted approach, aiming to restore stem‐stromal crosstalk, immune resolution, and vascular and neuromuscular communication. By treating the muscle‐resident cellular network as an interconnected system, we can better dismantle degenerative loops and promote homeostatic resilience in the aging muscle.

## Author Contributions

S.K. and M.J.K. contributed equally to this work. S.K. conceived the overall structure of the manuscript and conducted the literature review, drafting the initial version and preparing the figures and tables. M.J.K. contributed to revising the manuscript and improving clarity and organization. Y.R.Y. supervised the project, provided guidance on the conceptual direction, and critically revised the manuscript throughout the writing process. All authors reviewed and approved the final version of the manuscript.

## Funding

This work was supported by the National Research Foundation of Korea (NRF) grants funded by the Korean Government (RS‐2026‐25496234, RS‐2026‐25509438, and RS‐2026‐25514886), the National Research Council of Science & Technology (NST) grant funded by the Korean Government (MSIT) (CRC22015‐200), and the KRIBB Research Initiative Program.

## Conflicts of Interest

The authors declare no conflicts of interest.

## Data Availability

Data sharing not applicable to this article as no datasets were generated or analysed during the current study.
